# Authors’ reply

**Published:** 2011

**Authors:** Devdatta S Neogi, Kumar KV Ajay, Vivek Trikha, Chandra S Yadav

**Affiliations:** *Department of Orthopaedics, Jayprakash Narayan Apex Trauma Centre, All India Institute of Medical Sciences, Delhi, India*

Sir,

We have read the letter^1^ written in response to our case report and would like to respond in the following manner: Regarding the claim that this article is not beneficial at all to the audience of this prestigious journal, we would like to state that it is the most viewed and downloaded article of the issue.^2^

The authors of the letter failed to understand the reasons for performing plating along with acetabular fracture fixation. The authors preoperatively planned for the digastric osteotomy after reviewing the CT scans in detail. As reported in the article, the posterior wall fragment was superior in location and could not have been accessed adequately without the trochanteric digastric osteotomy. It helped in preventing the devitalization of the gluteus medius muscle by the otherwise strong retraction.^3^ Acetabular fracture requires anatomic reduction, and the digastrics osteotomy assisted in achieving the same. The fixation of this osteotomy prevented the placement of a nail for the fracture femur, which was already planned for, and hence the plating of femur.

We have discussed the rationale of using dynamic condylar screw along with antirotation screw for the reverse oblique fracture of the trochanter combined with neck femur fracture in the discussion. This case was operated nearly three years ago and at that time, the intramedullary devices as suggested by the authors of the letter were not available with us. Moreover, even now, such a nail is not considered ideal for the above indication even by the manufacturers themselves. We thank the authors for bringing the issue of varus which made us go back to the drawing board, and after reviewing the radiographs of both sides of hip joint, it was found that the difference was less than 5 degrees in both the hips. In all the cases in the literature also, the mode of treatment has been enumerated and most of the authors in different countries working under different work conditions have opted for the DCS with antirotation screw, which shows its versatility and also the benefits.^4^

We followed the worldwide accepted protocol for DVT prophylaxis^5^ hence did not mention in the article. We are thankful to the authors of this letter to give us an opportunity to make some points more clear.
